# Supplementary material for: Comprehensive patient-level classification and quantification of driver events in TCGA PanCanAtlas cohorts
Source: PLoS Genet. 2022 Jan 14;18(1):e1009996. doi: 10.1371/journal.pgen.1009996 (PMC8759692; doi:10.1371/journal.pgen.1009996)
Supplement: S4 Files — (ZIP) [file pgen.1009996.s004.zip › Aneuploidy/COHORTS GISTIC2/patient distributions/2021_11_23_15_0_CHOL_MALE.pdf]

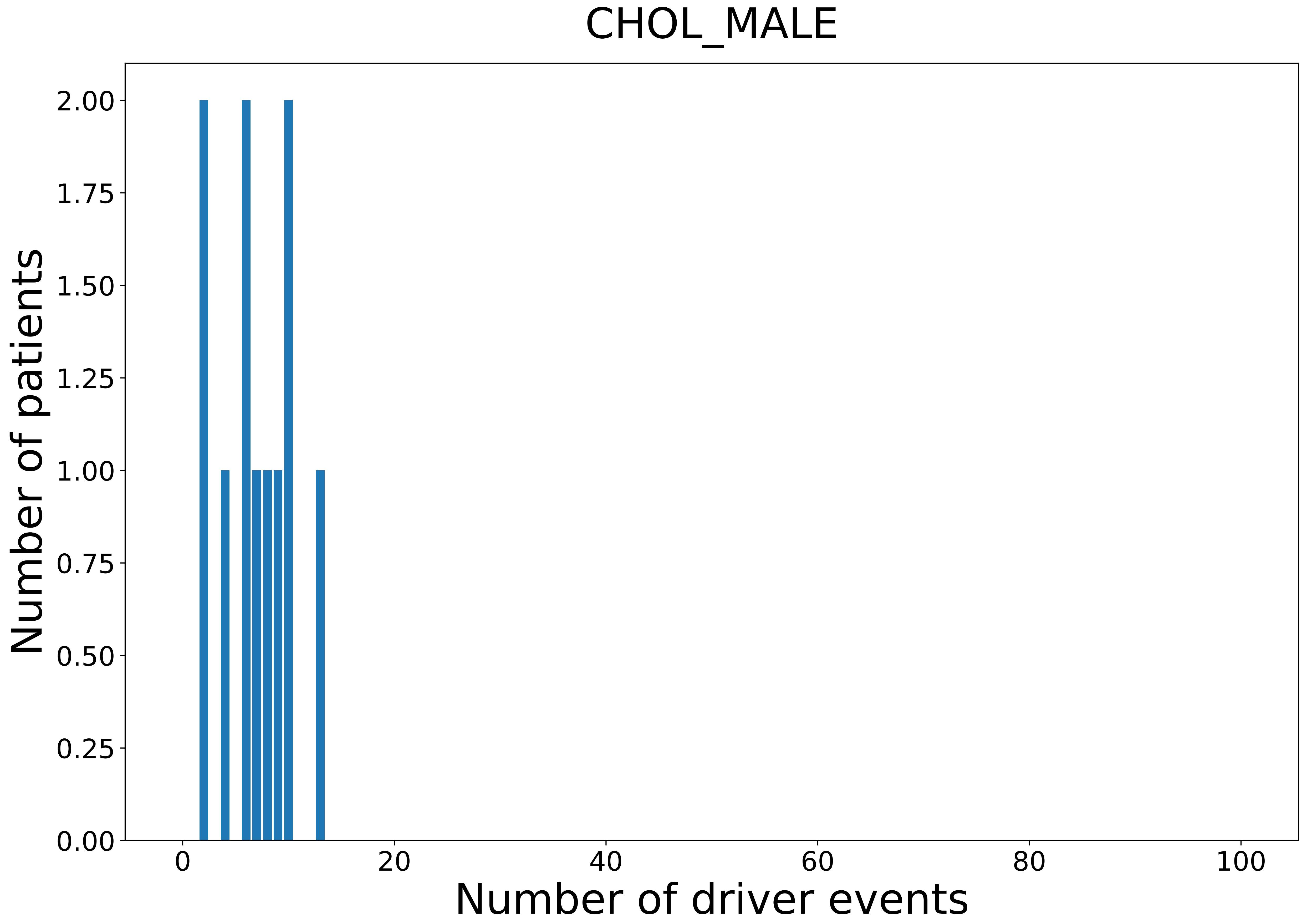
CHOL_MALE
Number of patients
Number of driver events
0.00
0.25
0.50
0.75
1.00
1.25
1.50
1.75
2.00
0
20
40
60
80
100
